# An autoinflammatory neurological disease due to interleukin 6 hypersecretion

**DOI:** 10.1186/1742-2094-10-29

**Published:** 2013-02-21

**Authors:** Ettore Salsano, Ambra Rizzo, Gloria Bedini, Loris Bernard, Valentina Dall’Olio, Sara Volorio, Monica Lazzaroni, Isabella Ceccherini, Dejan Lazarevic, Davide Cittaro, Elia Stupka, Rosina Paterra, Laura Farina, Mario Savoiardo, Davide Pareyson, Francesca L Sciacca

**Affiliations:** 1 UO Neurologia VIII, Department of Clinical Neurosciences, IRCCS Foundation, “C. Besta” Neurological Institute, Milan 20133, Italy; 2 Laboratory of Clinical Pathology and Medical Genetics, IRCCS Foundation, “C. Besta” Neurological Institute, Milan 20133, Italy; 3 Cellular Neurobiology Laboratory, Cerebrovascular Disease Unit, IRCCS Foundation, “C. Besta” Neurological Institute, Milan 20133, Italy; 4 IFOM, Fondazione Istituto FIRC di Oncologia Molecolare, Milan 20139, Italy; 5 Laboratory of Molecular Genetics, Istituto Giannina Gaslini, Genoa 16148, Italy; 6 Center for Translational Genomics and Bioinformatics, San Raffaele Scientific Institute, Milan 20132, Italy; 7 Unit of Neuroradiology, IRCCS Foundation, “C. Besta” Neurological Institute, Milan 20133, Italy

**Keywords:** Anakinra, Aseptic meningitis, *NLRP3* (*CIAS1*), Hearing loss, Interleukin-1, Interleukin-6, Leukoencephalopathy, *MEFV*, Tocilizumab

## Abstract

Autoinflammatory diseases are rare illnesses characterized by apparently unprovoked inflammation without high-titer auto-antibodies or antigen-specific T cells. They may cause neurological manifestations, such as meningitis and hearing loss, but they are also characterized by non-neurological manifestations. In this work we studied a 30-year-old man who had a chronic disease characterized by meningitis, progressive hearing loss, persistently raised inflammatory markers and diffuse leukoencephalopathy on brain MRI. He also suffered from chronic recurrent osteomyelitis of the mandible. The hypothesis of an autoinflammatory disease prompted us to test for the presence of mutations in interleukin-1−pathway genes and to investigate the function of this pathway in the mononuclear cells obtained from the patient. Search for mutations in genes associated with interleukin-1−pathway demonstrated a novel *NLRP3* (*CIAS1*) mutation (p.I288M) and a previously described *MEFV* mutation (p.R761H), but their combination was found to be non-pathogenic. On the other hand, we uncovered a selective interleukin-6 hypersecretion within the central nervous system as the likely pathogenic mechanism. This is also supported by the response to the anti-interleukin-6−receptor monoclonal antibody tocilizumab, but not to the recombinant interleukin-1−receptor antagonist anakinra. Exome sequencing failed to identify mutations in other genes known to be involved in autoinflammatory diseases. We propose that the disease described in this patient might be a prototype of a novel category of autoinflammatory diseases characterized by prominent neurological involvement.

## Background

Autoinflammatory diseases are rare monogenic or multifactorial (complex) illnesses, characterized by apparently unprovoked inflammation without high-titer auto-antibodies or antigen-specific T cells [1,2]. Autoinflammatory diseases may cause neurological manifestations, such as meningitis and hearing loss [3,4], but they are also characterized by non-neurological manifestations, such as cutaneous and articular symptoms and signs [1,5]. Among the already recognized autoinflammatory disorders are autosomal dominant diseases related to *NLRP3* (*CIAS1*) mutations, including chronic infantile neurologic cutaneous articular syndrome (CINCA) and Muckle-Wells syndrome (MWS), and the autosomal recessive (AR) familial Mediterranean fever (FMF) due to *MEFV* mutations. There are, however, patients with autoinflammatory phenotypes without known genetic mutations [2]. Here, we pinpoint an autoinflammatory disease due to selective IL-6 hypersecretion and characterized by predominant central nervous system (CNS) involvement.

## Case presentation

**Figure 1:**
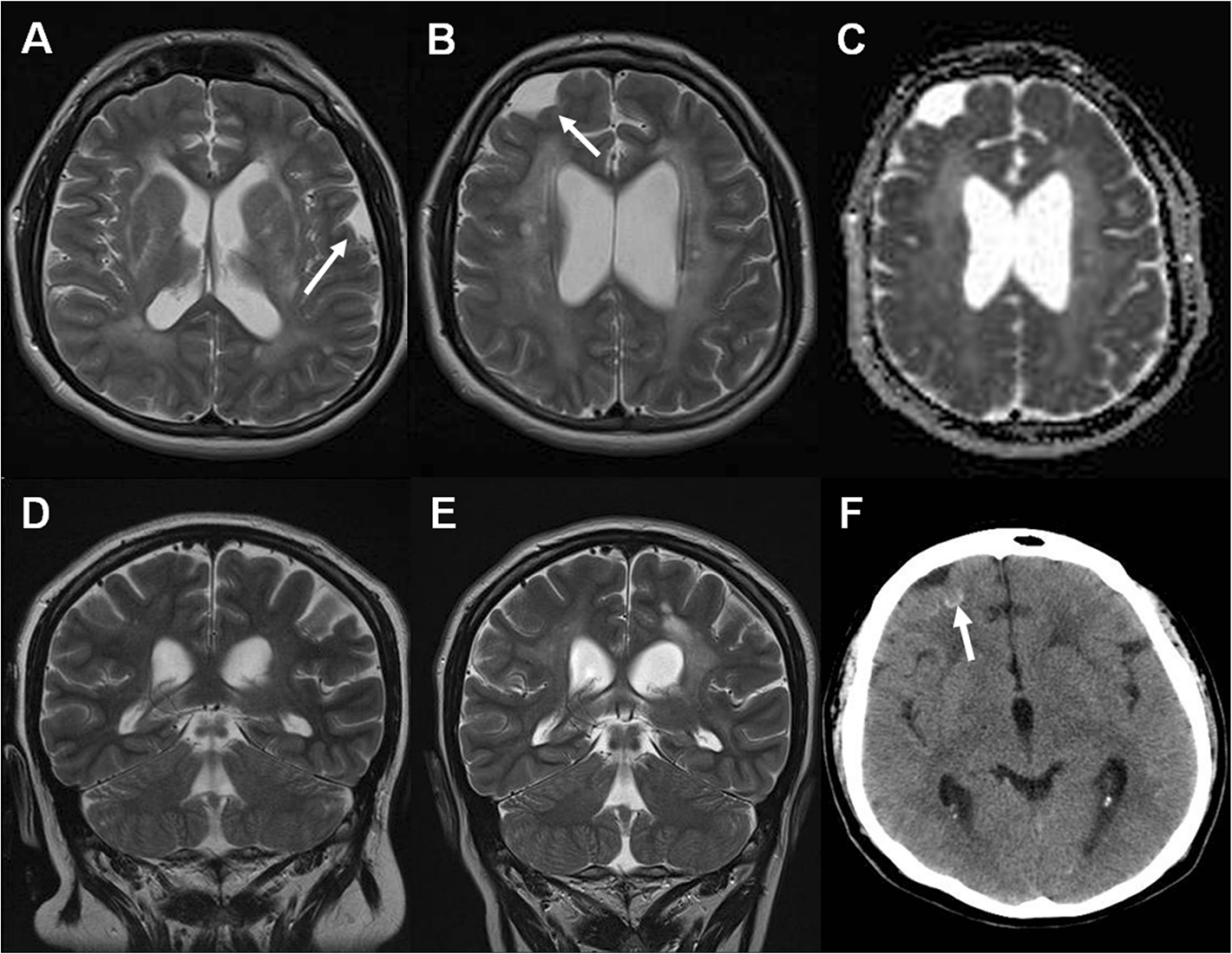
**Neuroradiological findings in different examinations.** Last magnetic resonance imaging (MRI) study (**A** and **B**, axial T2-weighted images; **C**, apparent diffusion coefficient (ADC) map) shows diffuse hyperintensities in the white matter, patchy involvement of the basal ganglia and thalami, and arachnoid cysts (arrows). ADC is increased, consistent with increased water content in the brain tissue. Comparison of MRI studies obtained 4 years apart (**D**, **E**, coronal T2-weighted images) shows progression of the leukoencephalopathy, decrease in size of the subarachnoid spaces along the convexity, and slight enlargement of the ventricles. Recent computed tomography (CT) scan (**F**) demonstrates tiny subcortical calcifications (arrows). See Additional file 1 for further details.

In March 2006, a 24-year-old Italian male patient was admitted for chronic headache (more severe in the morning), intermittent, low-grade fever and recurrent episodes of double vision that first occurred in March 2004. He also had a single tonic-clonic seizure in July 2004, for which no anti-epileptic therapy was started. His familial and past histories were unremarkable, except for chronic recurrent osteomyelitis of the mandible manifesting with repetitive mandibular pain and swelling, since the age of 20. Neurological examination revealed bilateral papilledema and increased deep tendon reflexes. No extra-neurological signs were present. Laboratory investigations revealed raised inflammatory markers and mild anemia. Brain magnetic resonance imaging (MRI) showed diffuse T2-weighted hyperintensity in the white matter, patchy involvement of the basal ganglia and thalami, and arachnoid cysts. Brain computed tomography (CT) showed tiny subcortical calcifications (Figure 1 and Additional file 1). Lumbar puncture revealed increased opening pressure (40 cmH_2_O), raised proteins and mild pleocytosis (see Table 1 for details). Despite extensive investigations (see Additional file 1), no infection was found and no autoimmune marker was demonstrated, except for fluctuating (negative to 1:640) titers of antinuclear antibodies (ANAs). Steroid therapy, started in October 2006, caused rapid clinical improvement with disappearance of headache, fever, papilledema, double vision, and mandibular osteomyelitis. However, headache reappeared when prednisone was tapered down to 30 mg/day, despite the co-administration of azathioprine 200 mg/day, and the patient developed progressive hearing loss, postural tremor and cushingoid features. Laboratory investigations still showed raised inflammatory markers, mild anemia, elevated cerebrospinal fluid (CSF) white-cell count (with a variable number of polymorphonuclear leukocytes), and proteinuria (1 g/24 h). Renal biopsy revealed only fusion of podocyte processes.

Chronic autoinflammatory disease was hypothesized, despite the lack of extra-neurological manifestations except for mandibular osteomyelitis.

**Table 1:** Key clinical features of our patient from 2004 to 2011

	**April 2004**^**1**^	**March 2006**	**September 2006**	**October 2006**	**September 2007**	**July 2008**	**November 2008**	**October 2009**	**September 2011**	**September 2011**
Clinical manifestations	Transient double vision	Intermittent fever, chronic headache (> morning), recurrent episodes of double vision, papilledema, one tonic-clonic seizure	Intermittent fever, chronic headache (> morning), recurrent episodes of double vision, papilledema	No symptom	No symptom	Morning headache, one episode of vomiting	Morning headache, hearing loss, cushingoid features	Morning headache, hearing loss, postural tremor, cushingoid features	Hearing loss, Babinski sign	Hearing loss, Babinski sign
Potentially disease-modifying therapy	No	No	No	Dexameth (8 mg/day × 7 days and, then, 4 mg/day × 7 days)	Prednisone 37.5 mg/day	Prednisone 30 mg/day, AZA 200 mg/day	Prednisone 35 mg/day	Prednisone 30 mg/day	Prednisone 12.5/day, Tocilizumab 800 mg/28 days	Methylpred 1,000 mg/day × 5 days
ESR (≤20 mm)	104	110	110	35	90	85	100	80	10	N/D
CRP (≤5 mg/L)	61	70.7	62	3	45	39	80	53	1	N/D
White Cell (%PMN; %L) (3,500 to 10,000/μL; 40 to 74%; 19 to 48%)	14,100 (67.4; 22)	12,410 (74.7; 14.1)	10,060 (75.1; 15.6)	18,240 (70.6; 21.1)	18,180 (90.5; 6.3)	12,610 (73.6; 17.5)	12,710 (77.1; 13.8)	13,680 (71.7; 19.9)	11,880 (78.5; 15.2)	N/D
Hemoglobin (14 to 16 g/dl)	11.7	10.6	10.1	11.8	12.8	11.4	11.5	11.7	15.8	N/D
IL-6 in serum (≤10 pg/ml)	N/A	N/A	N/A	N/A	44.23	22.4*	11.42	38.7	127	44.8
CSF pressure (5 to 15 cmH_2_O)	N/D	N/D	40	40	35	N/D	N/D	24	N/D	Normal
CSF White Cell/μl (L; PMN) (≤4/μl)	16 (N/D)	40 (25; 7)	40 (24; 12)	2.6	24 (12; 10)	45 (20; 12)	63 (10; 47)^2^	32 (26; 5)	65 (25; 22)	8 (7.3; 0.6)
CSF proteins (10 to 45 mg/dl)	63	84.1	98.4	33.0	73.9	90.6	98.2	123.2	102.2	54.5
CSF albumin / serum albumin × 1,000 (≤6.5)	6.37	12.36	15.51	8.37	12.08	18.26	21.18	20.59	16.86	11.12
Link IgG index (0.1 to 0.7); ReiberIgG index (≤0)	0.66; N/A	0.79; -5.07	0.96; 22.30	0.50; -21.95	1.90; 104.57	1.01; 25,61	1.09; 38.59	1.49; 118.81	0.77; -9.9	0.63; -14.89
OBs (negative)	No (type 1)	No (type 1)	No (type 1)	No (type 1)	No (type 1)	Yes (type 3)	No (type 4)	Yes (type 3)	No (type 4)	No (type 1)
IL-6 in CSF (≤10 pg/ml)	N/A	N/A	N/A	232.8	4544	N/A	9585	5389	7874	85
ANAs (negative)	N/D	1:320	1:640	1:640	1:40	1:320	1:80	1:80	1:40	N/D
Proteinuria (0 to 150 g/day)	N/D	N/D	N/D	N/D	800	1299	460	694	151	N/D
Other	Chronic recurrent osteomyelitis of the mandible			-	-	-	SSA 62.90 mg/l (nv ≤ 6.4)	Hepcidin 9.07 nmol/l (nv 4.3-7.06)	-	-

^1^Laboratory data obtained from another hospital.^2^The predominance of polymorphonuclear leukocytes (PMN) in this CSF sample helped us to hypothesize a possible autoinflammatory disease. The values shown in parentheses in the first column on the left are the normal values. ANAs, antinuclear antibodies; AZA, azathioprine; CRP, C-reactive protein; CSF, cerebrospinal fluid; Dexameth, dexamethasone; Methylpred, methylprednisone; ESR, erythrocyte sedimentation rate; IL-6, interleukin-6; L, lymphocytes; nv, normal value; N/A, Not Available; N/D, Not Done; OBs, oligoclonal bands; PMN, polymorphonuclear leukocytes; SSA, serum A amyloid protein.

## Methods

### DNA Sampling and genetic analyses

Genomic DNA for gene sequencing was isolated from peripheral blood mononuclear cells (PBMCs) using standard methods, and then stored at +4°C. Mutations in *NLRP3* (exon 3) [NM_001243133, RefSeq], *IL6* [NM_000600, RefSeq], *LIN28A* [NM_024674, RefSeq], *MEFV* (exons 1to 10) [NM_000243, RefSeq], *MIRLET7A3* [NR_029478, RefSeq]*, MVK* (exons 2, and 8 to 11) [NM_000431, RefSeq]*,* and *TNFRSF1A* (exons 2–4) [NM_001065, RefSeq] were searched for by direct sequencing. In particular, to search for mutations in the *IL6* gene, all six exons, including the 5^′^-UTR and 3^′^-UTR, and their flanking intron regions were amplified by the PCR method, purified and directly sequenced. Primers and PCR conditions are available on request. The c.864C>G (p.I288M) mutation in *NLRP3* and the c.2282G>A (p.R761H) mutation in *MEFV* were also searched for in the patient’s family members (that is, the father, mother, two sisters and one brother) by direct sequencing.

### Exome analysis

To prepare a next-generation sequencing library, genomic DNA was extracted from PBMCs, and randomly fragmented by a Covaris Adaptive Focused Acoustics™ (AFA) instrument to obtain an average fragment size of 200 to 300 base pairs. Then, the genomic DNA fragments were end-repaired, adenylated, linked to Illumina-specific adapter sequences, and amplified by PCR. Exome capture was performed using the TruSeq™ Exome Enrichment Kit (Illumina, San Diego, CA, USA). Sequencing was performed on the Illumina Genome Analyzer HiSeq2000 following the manufacturer’s instructions. The average coverage was 20-fold. The sequencing reads were aligned to the human reference genome hg19 using the Burrows-Wheeler Aligner (BWA) software. We analyzed the data using the Genome Analysis Toolkit (GATK) variant pipeline with respect to 1,000 Genomes Data, Single Nucleotide Polymorphism Database (dbSNP) and in-house data, and we used the SnpEff software to predict the effects of sequence variants.

### Body fluid collection

CSF and/or serum of the patient, patient family members and unrelated controls were used for laboratory analyses on the same day of the sampling or were stored at −40°C in the serum and CSF bank of the Neurological Institute ‘Carlo Besta’. Control CSF was obtained from patients with non-inflammatory neurological diseases (that is, brain tumors, neurodegenerative and heredo-metabolic diseases).

### Determination of cytokines and IL-6-R

IL-1β and soluble IL-6R (sIL-6R) were determined by ELISAs (Human IL-1β/IL-1F2 Quantikine ELISA Kit, catalogue number DLB50, and Human IL-6 R alpha Quantikine ELISA Kit, catalogue number DR600; R&D Systems, Minneapolis, MN, USA; http://www.rndsystems.com). In addition, CSF IL-1β was determined by a high sensitivity immunoassay (Human IL-1β/IL-1F2 Quantikine HS Immunoassay, catalogue number HSLB00C, R&D Systems). CSF2/GM-CSF, IFN-γ, IL-6, IL-8, IL-17A, and TNF-α were determined by the Human Inflammatory Cytokines Multi-Analyte ELISArray Kit from SABiosciences (catalogue number MEH-004A; SABiosciences, Frederick, MD, USA; http://www.sabiosciences.com), and IL-8 and TNF-α concentrations were also determined by ELISA (GE Healthcare Europe GmbH, Milan, Italy; http://www.gelifesciences.com). IL-6 concentrations were determined by an electrochemiluminescent immunoassay (IL6 Elecsys, catalogue number 05109442190, Roche Diagnostics, Mannheim, Germany; http://www.roche.de) performed using a Cobas E601 analyzer (Roche Diagnostics), whereas the level of *IL-6* mRNA was investigated by real-time PCR using standard methods, and *18S* as housekeeping gene. In the monocytes of the patient and ten healthy controls, membrane-bound IL-6R was roughly quantified by western blot using an anti-human IL-6 Rα antibody (Human IL-6 R alpha Affinity Purified Polyclonal Ab, Goat IgG, AF-227-NA, R&D Systems) and anti-beta-actin antibody as internal control (Monoclonal Anti-β-Actin antibody produced in mouse-clone AC-15, purified immunoglobulin, buffered aqueous solution, catalogue number A1978, Sigma-Aldrich, Milan, Italy; http://www.sigmaaldrich.com).

### Cell cultures

Monocytes were obtained from the peripheral blood of the patient, the patient’s family members, and healthy controls. In order to determine the IL-1β and IL-6 secretion *in vitro*, monocytes were cultured in RPMI Medium 1640 (Invitrogen, Carlsbad, CA, USA; http://www.invitrogen.com) containing 10% fetal bovine serum (FBS, Gibco – Invitrogen, Carlsbad, CA, USA) for approximately 18 hours at 37°C/5%CO_2_, and then they were activated by 1 μg/ml of lipopolysaccaride (LPS, catalogue number L6529, Sigma-Aldrich) for 3 hours at 37°C in fresh RPMI Medium 1640. For the evaluation of the sole IL-1β secretion, the monocytes were subsequently incubated in the presence of 1 m*M* ATP (catalogue number A6419, Sigma-Aldrich) for an additional 15 minutes. Supernatants were collected for quantification of IL-1β and IL-6; for the evaluation of *IL-6* mRNA levels, RNA was isolated from the monocytes using the QIAamp RNA Blood Mini Kit (catalogue number 52304; Qiagen, Milan, Italy; http://www.qiagen.com); for the evaluation of membrane-bound IL-6R, proteins were isolated using Laemmli Buffer.

### Statistical analyses

Statistical analyses were performed using GraphPad Prism 5.00 for Windows, GraphPad Software, San Diego California USA, http://www.graphpad.com.

### Ethical approval and patient consent

Anakinra was administered off-label, the experimental treatment with tocilizumab was approved by the independent ethics committee of the IRCCS Foundation, C. Besta Neurological Institute, and the patient provided written informed consent for both treatments.

## Results

**Figure 2:**
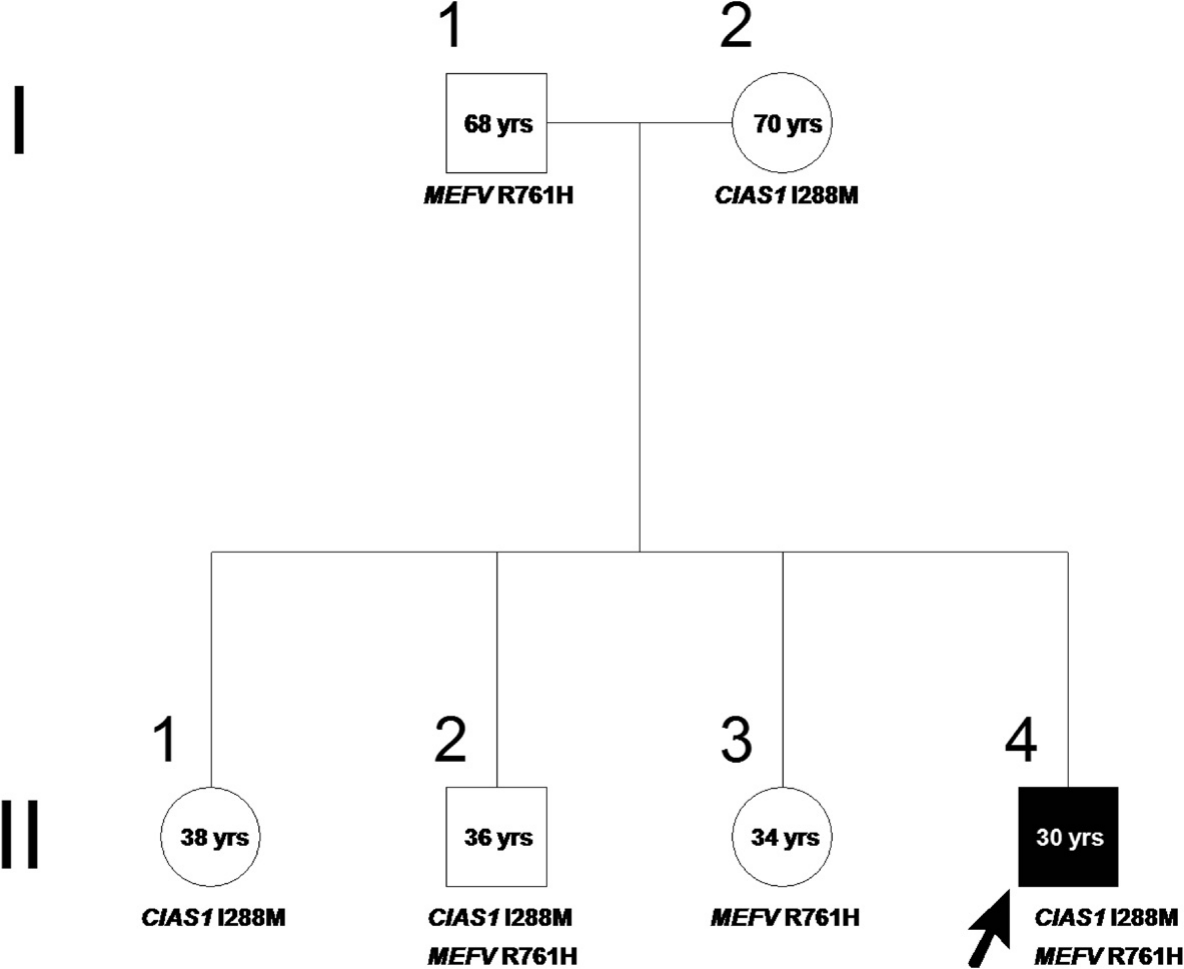
**Absence of co-segregation of the mutations I288M in*NLRP3*(*CIAS1*) and R761H in*MEFV*with the disease phenotype in the patient’s family members.**

**Figure 3:**
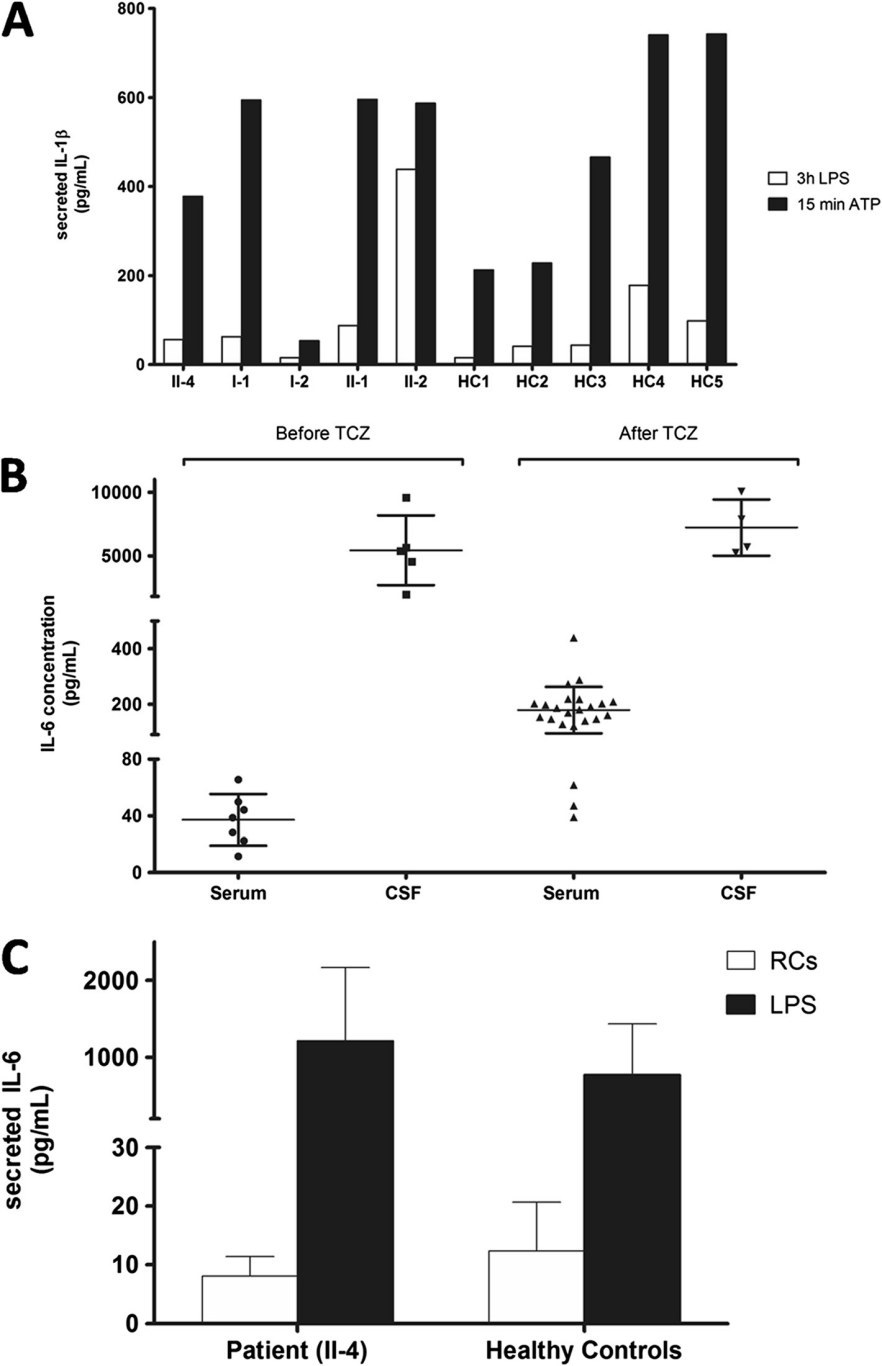
***In vitro *IL-1β secretion, serum and cerebrospinal fluid (CSF) IL-6 in the patient**, **and *in vitro *IL-6 secretion.** (**A**) IL-1β secretion from monocytes of the proband (II-4), his family members (I-1, I-2, II-1 and II-2) and five unrelated healthy controls (HC1-5) after 3 hours of LPS incubation, or 15 minutes of ATP exposure following LPS stimulation, was quantified by ELISA. Results are expressed as pg/ml per 10^6^ cells. For the proband the values are the mean of three experiments. Unlike monocytes from patients bearing a pathogenic *NLRP3* mutation [8], monocytes from the proband and his family members, harboring the p.I288M *NLRP3* (I-2 and II-1), p.R761H *MEFV* (I-1), or both (II-2 and II-4) mutations, displayed a pattern of IL-1β secretion similar to healthy controls, and characterized by further IL-1β secretion following ATP induction. (**B**) Serum and CSF IL-6 in our patient before and under tocilizumab (TCZ). Before tocilizumab, IL-6 concentration was evaluated in patient’s serum samples (n=7) and CSF samples (n=5), each obtained ≥4 weeks apart. IL-6 was raised in serum (37.20±18.17, normal value (nv) ≤10 pg/ml) and CSF (5432±2742, nv ≤10) despite a high dose of prednisone. CSF IL-6 concentration was very high, and the mean ratio of CSF IL-6/serum IL-6 was about 146, suggesting that IL-6 is produced in central nervous system (CNS). Under tocilizumab (and prednisone reduction ), serum IL-6 concentration significantly increased (178.8±84.14 (n=23) versus 37.20±18.17 (n=7), *P*= 0.0003; Mann–Whitney test); the slight increase of CSF IL-6 concentration was not significant (7221±2212 (n=4) versus 5432±2742 (n=5), *P*= 0.2857; Mann–Whitney test). (**C**) *In vitro* IL-6 secretion. IL-6 amounts from monocytes of our patient (Pt) and 13 healthy controls (HCs) are similar under resting conditions (RCs; 8.043±3.343 pg/ml (n=7) versus 12.36±8.3) and after LPS stimulation (LPS; 1209±956.6 pg/ml (n=7) versus 772.7±663.2), further suggesting that the deregulation of IL-6 production occurs primarily in CNS. See Additional file 2 for *IL-6* mRNA level analysis.

A novel c.864C>G (p.I288M) *NLRP3* missense mutation and the previously described c.2282G>A (p.Arg761His) *MEFV* mutation were found. No mutation was found in the *TNFRSF1A* and *MVK* genes, which are associated with other autoinflammatory diseases, and karyotype and array-CGH analysis (see Additional file 1) were normal [1,6]. The p.I288M mutation - not reported in the INFEVERS registry (http://fmf.igh.cnrs.fr/ISSAID/infevers/) - is located in exon 3, where most of the pathogenic *NLRP3* mutations are located; the isoleucine residue at position 288 is conserved in mammals and birds, and its substitution with methionine is non-conservative. Prediction using the sorting intolerant from tolerant (SIFT) software indicates that the p.I288M substitution is of probable functional importance to the protein. Moreover, only one *MEFV* mutation was found in a subset of FMF patients, although FMF is traditionally considered an autosomal recessive disease [7], and both *NLRP3*-related diseases and FMF are IL-1β activation disorders, which may cause aseptic meningitis [3,4]. Hence, the combination of these two mutations could actually be pathogenic. However, segregation analysis in the proband’s family showed that neither the p.I288M *NLRP3* mutation alone nor the combination of the two mutations segregated with the phenotype (Figure 2). Moreover, i) there was no response to the IL-1 receptor antagonist anakinra (Amgen Inc), which is efficacious in IL-1β activation disorders; ii) circulating IL-1β concentration was similar in the serum of the patient (0.19 ± 0.3 pg/ml, n=12) and ten unrelated healthy controls (0.16±0.3 pg/ml); iii) no IL-β was detected in the CSF of the patient, even with a high sensitivity immunoassay; iv) no difference in IL-1β secretion was observed comparing patient and control monocytes under resting conditions and after LPS alone or LPS followed by ATP stimulation (Figure 3A) [8]. Therefore, atypical IL-1β activation disorders were forcibly excluded.

Therefore, we measured CSF2/GM-CSF, IFN-γ, IL-6, IL-8, IL-17α, and TNF-α in the patient’s serum samples, and we found that, among these cytokines, only IL-6 was increased. Indeed, the IL-6 concentration evaluated in seven independent samples of the patient’s serum, each obtained ≥4 weeks apart, was 37.20 ± 18.17 pg/ml (nv ≤10 pg/ml). On the basis of this finding, and given that the patient has been complaining of neurological symptoms, we also measured IL-6 in patient’s CSF samples, and we found that its concentration was much higher than in serum. Indeed, the CSF IL-6 concentration evaluated in five independent samples of the patient’s CSF, each obtained ≥4 weeks apart, was 5,432 ± 2,742 pg/ml (nv ≤10 pg/ml) (Figure 3B) [9]. For comparison, the CSF IL-6 concentration evaluated in 17 patients with non-inflammatory neurological diseases (that is,thirteen with brain tumors and four with neurodegenerative diseases) was 10.4 ± 7.8 pg/ml, with a value ≤10 pg/ml in eleven of seventeen patients. Finally, we measured both IL-8 and TNF-α in at least one patient’s CSF sample, and we found that TNF-α was undetectable, whereas IL-8 was increased (490.3 pg/ml, nv<32), probably because IL-8 is induced by IL-6 from CSF mononuclear cells [10]. Overall, these data suggest that IL-6 played a key role in the pathogenesis of the disease under investigation.

As a consequence, the patient was treated with the anti-IL-6 receptor (IL-6R) monoclonal antibody tocilizumab (Roche), 8 mg/kg every 28 days. Ten days after the first tocilizumab administration, the blood inflammatory markers normalized (Table 1). Moreover, the steroid intake was rapidly reduced without worsening of symptoms. Progressive disappearance of cushingoid features and postural tremor was observed and the patient definitely stopped complaining about headache after dividing the daily prednisone dose (12.5 mg/day) between morning (7.5 mg/day) and evening (5 mg/day). However, CSF parameters remained abnormal, and serum IL-6 concentration, which was evaluated in 23 independent samples of the patient’s serum (each obtained about 4 weeks apart), markedly increased to 178.8±84.14 pg/ml (nv ≤10.0pg/ml) (Figure 3B). This increase is probably due to the inhibition of the clearance of IL-6 from serum by tocilizumab. In fact, the main elimination pathway of IL-6 from serum may be from the binding of IL-6 to IL-6R, including the soluble form. As a consequence, IL-6 may accumulate in serum when the anti-IL-6R monoclonal antibody tocilizumab binds to the IL-6-binding site of its receptor [11]. In contrast, no significant change in the IL-6 concentration was found in four independent samples of the patient’s CSF (mean value 7221±2212 pg/ml) (Figure 3B), but we detected a reduction of CSF IL-8 concentration in two independent samples of the patient’s CSF (from 490.3 pg/ml before tocilizumab to 157.0 pg/ml and, then, 53.0 pg/ml (nv<32.0) under tocilizumab). This reduction suggests that tocilizumab may diffuse into CSF, and inhibit the IL-6-mediated effects (though slightly).

The high CSF IL-6/serum IL-6 ratio suggests that IL-6 is produced in the CNS and/or CSF, from which it diffuses into blood (Figure 3B and Table 1). To further corroborate this hypothesis, the IL-6 secretion between patient and control monocytes under resting conditions and after activation with LPS was compared, and no significant difference was observed (Figure 3C). Moreover, no clear-cut difference was found between the levels of *IL-6* mRNA after LPS stimulation (see Additional file 2).

To identify the cause of the chronic IL-6 hypersecretion, we searched for mutations in the *IL6* gene, including its 5^′^- and 3^′^-UTRs, and in *LIN28A* and *MIRLET7A3*, which encode for molecules involved in the IL-6 regulatory pathway [12], with no mutation found. We ruled out mutations in the *LPIN2*, *PSMB8* and *SH3BP2* genes by using exome analysis, despite these genes having been associated with autoinflammatory diseases with different phenotypes. We investigated the serum concentration of soluble IL-6R (sIL-6R), which mediates the IL-6 clearance, but no clear-cut abnormality was found before or under tocilizumab (see Additional file 3) [11]. Moreover, we found that the amount of membrane-bound IL-6R in the patient’s monocytes was similar to that of healthy controls (not shown). Finally, there was no infection by Human Herpes Virus 8 (HHV8), which contains an *IL-6* homologous gene [13].

## Discussion

We report a case of chronic inflammatory disease, which can be considered as the prototype of a novel category of autoinflammatory diseases characterized by primary CNS involvement. The key manifestations are chronic meningitis, progressive hearing loss, diffuse white-matter hyperintensity, and persistently raised inflammatory markers. The pathogenic mechanism of this disease is a selective and chronic IL-6 hypersecretion. Indeed, no other cytokine among those investigated was consistently increased in CSF and serum, and the anti-IL-6R monoclonal antibody tocilizumab was partially able to counteract the phenotype. The persistence of abnormal CSF parameters is likely due to the low permeability of the blood brain barrier for tocilizumab.

Despite extensive laboratory investigations, the cause of IL-6 hypersecretion was not identified. Although patients suffering from autoinflammatory diseases (AID) who are double heterozygotes for mutations in two known AID-associated genes have been reported [14], it is amazing that we found two mutations in known AID-associated genes (*NLRP3* and *MEFV*) that were proved to be unrelated to the disease under investigation. However, we cannot exclude that the p.I288M *NLRP3* variant and the p.R761H *MEFV* mutation contribute to the inflammatory phenotype in the presence of other genetic or permissive environmental factors triggering IL-6 hypersecretion [2]. Microglia, astroglia, or both, are the likely source of IL-6 as suggested by IL-6 CSF/serum ratio [5], and by the lack of abnormal IL-6 hypersecretion by patient monocytes *in vitro*. The condition of our patient, therefore, might be analogous to the neurological disease induced in transgenic mice by IL-6 cerebral overexpression [15,16].

The CNS hypersecretion of IL-6 may cause: 1) an increased blood–brain barrier permeability, leading to subtle edema. This may explain why there are no focal or diffuse CNS symptoms, and the somatosensory and motor-evoked potentials are near normal (see Additional file 1), despite extensive MRI abnormalities; 2) CSF inflammation with increased intracranial pressure, and 3) inner ear damage [5,17]. Moreover, IL-6 diffusion to the blood compartment may cause the increase of inflammatory markers and chronic anemia (through hepcidin increase), fusion of the feet of podocytes, and proteinuria. Finally, it should be noted that chronic recurrent multifocal osteomyelitis and cherubism are both autoinflammatory diseases [1], suggesting that the chronic recurrent osteomyelitis of the mandible observed in our case may be expression of an autoinflammatory state.

ANA positivity with titer fluctuations (up to 1:640) may suggest a role of autoimmunity in the pathogenesis of the disease, with proportions differing at different phases [18]. However, ANAs may be present in healthy subjects and patients with a variety of diseases, including non-rheumatic diseases [19], and their level may be increased by IL-6 [20]. Therefore, in our case, ANA positivity might be a clinically non-specific finding related to the chronic inflammation.

Chronic monogenic autoinflammatory diseases, including CINCA, autoimmune diseases including neurolupus, and complex diseases with no specific antigen/antibody but with evidence of cellular inflammation, such as neuro-Behçet disease and primary angiitis of the CNS, may cause chronic aseptic meningitis, with or without evidence of diffuse white matter abnormalities on brain MRI [5,21-23]. In our case, however, the criteria proposed for the diagnosis of these diseases are not satisfied despite long-term follow up, and the pattern of cytokine production is peculiar. In particular, CNS systemic lupus erythematosus was excluded on the basis of the American College of Rheumathology criteria because of the absence of a wide range of extra-neurological manifestations, such as arthritis, dermatitis, and glomerulonephritis, and because of the presence of persistently raised inflammatory markers, leukocytosis and slightly increased (or normal) complement levels (see Additional file 1) [24]. Neuro-Behçet disease was excluded because of the absence of symptoms consistent with focal or multifocal CNS dysfunction, and absence of extra-neurological features, namely, oral and genital ulcerations and eye and skin lesions. Moreover, HLA-B*51 was absent, the levels of other pro-inflammatory cytokines were normal [25], and no response to anakinra was obtained [26]. Primary angiitis of the CNS was excluded because of lack of neurological deficit, presence of constantly raised inflammatory markers, and young age at onset [27]. Infectious diseases were also excluded since there was no worsening after the long corticosteroid and immunosuppressive treatment. Castleman disease, a rare disorder biologically characterized by raised serum IL-6 levels, was excluded on the basis of clinical features, unremarkable total-body (18F)-fluorodeoxyglucose-positron emission tomography/computed tomography, and absence of serum HHV8 DNA and anti-HHV8 antibodies [12].

## Conclusions

This report demonstrates a case of autoinflammatory neurological disease due to IL-6 hypersecretion with aseptic chronic meningitis as the key feature. We suggest that all patients with non-infective chronic meningitis of unknown etiology should be investigated for IL-6 hypersecretion.

## Consent

Written informed consent was obtained from the patient for publication of this case report and any accompanying images. A copy of the written consent is available for review by the Editor-in-Chief of this journal.

## Abbreviations

ADC: Apparent diffusion coefficient; AID: Autoinflammatory disease; ANAs: Antinuclear antibodies; AR: Autosomal recessive; CINCA: Chronic infantile neurologic cutaneous articular syndrome; CNS: Central nervous system; CSF: Cerebrospinal fluid; CT: Computed tomography; ELISA: Enzyme-linked immunosorbent assay; FBS: Fetal bovine serum; FMF: Familial Mediterranean fever; HC(s): Healthy control(s); HHV8: Human herpes virus 8; IL-6: Interleukin-6; IL-6R: Interleukin-6 receptor; LPS: Lipopolysaccharide; MRI: Magnetic resonance imaging; MWS: Muckle-Wells syndrome; nv: Normal value; PACNS: Primary angiitis of the central nervous system; PBMCs: Peripheral blood mononuclear cells; PCR: Polymerase chain reaction; Pt: Patient; RCs: Resting conditions; sIL-6R: Soluble interleukin-6 receptor; TCZ: Tocilizumab; TNF: Tumor necrosis factor; VEGF: Vascular endothelial growth factor.

## Competing interests

The authors declare that they have no competing interests.

## Authors’ contributions

ESa took the lead in drafting the manuscript and has made substantial contributions to study concept and design, acquisition, analysis and interpretation of data, statistical analysis and study coordination. AR, GB and ML carried out determination of cytokines and IL6-R, and performed cell culture experiments. LB, VD, SV and IC carried out sequence analyses and real-time PCR studies. DL, DC, ESt and RP carried out exome analysis and subsequent sequence analyses. LF has been involved in drafting the manuscript, and has made substantial contributions to acquisition, analysis and interpretation of MRI data. MS and DP have been involved in revising the manuscript critically for important intellectual content and have made substantial contributions to analysis and interpretation of clinical and MRI data. FLS has been involved in drafting the manuscript and in revising it critically for important intellectual content, has made substantial contributions to study concept and design, and to analysis and interpretation of laboratory data. All authors read and approved the final manuscript.

## Supplementary Material

Additional file 1 Neuroradiological, electrophysiological and laboratory data. Click here for file

Additional file 2 Figure showing IL-6 mRNA levels. Click here for file

Additional file 3 Figure showing soluble IL-6 receptor concentrations before and after tocilizumab. Click here for file

## References

[B1] Masters SL Simon A Aksentijevich I Kastner DL Horror autoinflammaticus: the molecular pathophysiology of autoinflammatory disease Annu Rev Immunol 2009 27 621 668 10.1146/annurev.immunol.25.022106.141627 19302049 PMC2996236

[B2] Aksentijevich I Kastner DL Genetics of monogenic autoinflammatory diseases: past successes, future challenges Nat Rev Rheumatol 2011 7 469 478 10.1038/nrrheum.2011.94 21727933

[B3] Karachaliou I Karachalios G Charalabopoulos A Charalabopoulos K Meningitis associated with familial Mediterranean fever Int J ClinPractSuppl 2005 147 60 61 10.1111/j.1368-504x.2005.00290.x 15875625

[B4] Kitley JL Lachmann HJ Pinto A Ginsberg L Neurologic manifestations of the cryopyrin-associated periodic syndrome Neurology 2010 74 1267 1270 10.1212/WNL.0b013e3181d9ed69 20404307

[B5] Goldbach-Mansky R Dailey NJ Canna SW Gelabert A Jones J Rubin BI Kim HJ Brewer C Zalewski C Wiggs E Hill S Turner ML Karp BI Aksentijevich I Pucino F Penzak SR Haverkamp MH Stein L Adams BS Moore TL Fuhlbrigge RC Shaham B Jarvis JN O'Neil K Vehe RK Beitz LO Gardner G Hannan WP Warren RW Horn W Neonatal-onset multisystem inflammatory disease responsive to interleukin-1beta inhibition N Engl J Med 2006 355 581 592 10.1056/NEJMoa055137 16899778 PMC4178954

[B6] Reddy S Jia S Geoffrey R Lorier R Suchi M Broeckel U Hessner MJ Verbsky J An autoinflammatory disease due to homozygous deletion of the IL1RN locus N Engl J Med 2009 360 2438 2444 10.1056/NEJMoa0809568 19494219 PMC2803085

[B7] Booty MG Chae JJ Masters SL Remmers EF Barham B Le JM Barron KS Holland SM Kastner DL Aksentijevich I Familial Mediterranean fever with a single MEFV mutation: where is the second hit? Arthritis Rheum 2009 60 1851 1861 10.1002/art.24569 19479870 PMC2753538

[B8] Gattorno M Tassi S Carta S Delfino L Ferlito F Pelagatti MA D'Osualdo A Buoncompagni A Alpigiani MG Alessio M Martini A Rubartelli A Pattern of interleukin-1beta secretion in response to lipopolysaccharide and ATP before and after interleukin-1 blockade in patients with CIAS1 mutations Arthritis Rheum 2007 56 3138 3148 10.1002/art.22842 17763411

[B9] Kestner M Rosler AE Baumgärtner M Lindner A Orth M CSF interleukin 6 – a useful biomarker of meningitis in adults?/Liquor Interleukin 6 – einsinnvoller Biomarker für die Meningitis beimErwachsenen Laboratoriums Medizin 2011 35 107 113

[B10] Hashizume M Higuchi Y Uchiyama Y Mihara M IL-6 plays an essential role in neutrophilia under inflammation Cytokine 2011 54 92 99 10.1016/j.cyto.2011.01.007 21292497

[B11] Nishimoto N Terao K Mima T Nakahara H Takagi N Kakehi T Mechanisms and pathologic significances in increase in serum interleukin-6 (IL-6) and soluble IL-6 receptor after administration of an anti-IL-6 receptor antibody, tocilizumab, in patients with rheumatoid arthritis and Castleman disease Blood 2008 112 3959 3964 10.1182/blood-2008-05-155846 18784373

[B12] Iliopoulos D Hirsch HA Struhl K An epigenetic switch involving NF-kappaB, Lin28, Let-7 MicroRNA, and IL6 links inflammation to cell transformation Cell 2009 139 693 706 10.1016/j.cell.2009.10.014 19878981 PMC2783826

[B13] El-Osta HE Kurzrock R Castleman’s disease: from basic mechanisms to molecular therapeutics Oncologist 2011 16 497 511 10.1634/theoncologist.2010-0212 21441298 PMC3228122

[B14] Singh-Grewal D Chaitow J Aksentijevich I Christodoulou J Coexistent MEFV and CIAS1 mutations manifesting as familial Mediterranean fever plus deafness Ann Rheum Dis 2007 66 1541 10.1136/ard.2007.075655 17934081 PMC2111617

[B15] Campbell IL Abraham CR Masliah E Kemper P Inglis JD Oldstone MB Mucke L Neurologic disease induced in transgenic mice by cerebral overexpression of interleukin 6 Proc Natl Acad Sci USA 1993 90 10061 10065 10.1073/pnas.90.21.10061 7694279 PMC47713

[B16] Brett FM Mizisin AP Powell HC Campbell IL Evolution of neuropathologic abnormalities associated with blood–brain barrier breakdown in transgenic mice expressing interleukin-6 in astrocytes J Neuropathol Exp Neurol 1995 54 766 775 10.1097/00005072-199511000-00003 7595649

[B17] Wakabayashi K Fujioka M Kanzaki S Okano HJ Shibata S Yamashita D Masuda M Mihara M Ohsugi Y Ogawa K Okano H Blockade of interleukin-6 signaling suppressed cochlear inflammatory response and improved hearing impairment in noise-damaged mice cochlea Neurosci Res 2010 66 345 352 10.1016/j.neures.2009.12.008 20026135

[B18] Nishimura H Strominger JL Involvement of a tissue-specific autoantibody in skin disorders of murine systemic lupus erythematosus and autoinflammatory diseases ProcNatlAcadSci USA 2006 103 3292 3297 10.1073/pnas.0510756103 PMC1413900 16492738

[B19] Solomon DH Kavanaugh AJ Schur PH American College of Rheumatology Ad Hoc Committee on Immunologic Testing Guidelines: Evidence-based guidelines for the use of immunologic tests: antinuclear antibody testing Arthritis Rheum 2002 47 44 434 10.1002/art1.10246 12209492

[B20] Illei GG Shirota Y Yarboro CH Daruwalla J Tackey E Takada K Fleisher T Balow JE Lipsky PE Tocilizumab in systemic lupus erythematosus: data on safety, preliminary efficacy, and impact on circulating plasma cells from an open-label phase I dosage-escalation study Arthritis Rheum 2010 62 542 552 10.1002/art.27221 20112381 PMC3057537

[B21] Cohen BA Chronic meningitis Curr Neurol Neurosci Rep 2005 5 429 439 10.1007/s11910-005-0030-3 16263053

[B22] Prabhakaran S Bramlage M Edgar MA Diamond B Hardin JA Volpe BT Overwhelming leukoencephalopathy as the only sign of neuropsychiatric lupus J Rheumatol 2005 32 1843 1845 16142887

[B23] Ginsberg L Kidd D Chronic and recurrent meningitis Pract Neurol 2008 8 348 361 10.1136/jnnp.2008.157396 19015295

[B24] Joseph FG Scolding NJ Neurolupus Pract Neurol 2010 10 4 15 10.1136/jnnp.2009.200071 20130291

[B25] Hamzaoui K Hamzaoui A Guemira F Bessioud M Hamza M Ayed K Cytokine profile in Behçet’s disease patients. Relationship with disease activity Scand J Rheumatol 2002 31 205 210 10.1080/030097402320318387 12369651

[B26] Botsios C Sfriso P Furlan A Punzi L Dinarello CA Resistant Behçet disease responsive to anakinra Ann Intern Med 2008 149 284 286 18711165 10.7326/0003-4819-149-4-200808190-00018

[B27] Birnbaum J Hellmann DB Primary angiitis of the central nervous system Arch Neurol 2009 66 704 709 10.1001/archneurol.2009.76 19506130

