# Supplementary material for: An autoinflammatory neurological disease due to interleukin 6 hypersecretion
Source: J Neuroinflammation. 2013 Feb 21;10:29. doi: 10.1186/1742-2094-10-29 (PMC3601972; doi:10.1186/1742-2094-10-29)
Supplement: Additional file 1 — Neuroradiological, electrophysiological and laboratory data. [file 1742-2094-10-29-S1.doc]

**SUPPLEMENTARY DATA**

**Brain MRI and CT studies**

From 2006 to 2011 the patient was submitted to nine MR studies including post-contrast examinations, DWI, and ^1^H-MRS. There was a moderate progression through the years in the extension and conspicuity of the signal changes, but the pattern and distribution of the abnormalities remained essentially unchanged. In addition, from 2006 to 2011, a mild increase in the ventricular size and a decrease in the size of the cerebral sulci occurred, consistent with a possible impairment of CSF circulation. Finally, comparison of the apparent diffusion coefficient (ADC) images of 2006 and 2011 demonstrated that there was an increase in the value of ADC, consistent with increased water content in the brain tissue.

The findings observed in the last examination will be now described in detail: The 3^rd^ and the lateral ventricles are enlarged, with mild prevalence of the left lateral ventricle. The sulci are visible; on the right and left frontal convexities there are three small arachnoid cysts (which might be due to entrapment of CSF by arachnoid adhesions). In the posterior fossa, signal changes consist in T2 hyperintensity in the hilum of the dentate nuclei. These nuclei are slightly hyperintense in T1-weighted images. A tiny paramedian pontine abnormality is also observed, consistent with lacunar infarction. Signal abnormalities are more conspicuous in the basal ganglia and white matter. The basal ganglia are depicted by thin bands of abnormal signal intensities in the external capsule, on the lateral margin of the posterior limb of the internal capsule, and also in the laminae separating the putamen from the pallidum and the external from the internal pallidum (lateral and medial medullary laminae). The internal pallida are relatively hypointense in T2-weighted images. The white matter abnormalities involve the deep and periventricular frontal and parieto-occipital regions with sparing of the subcortical fibers. In the affected white matter, a few focal areas with more marked hyperintense signal are present. A few millimetres from the margin of the frontal horns and the bodies of the lateral ventricles, a thin band of hypointensity in T2-weighted images, possibly representing the superior fronto-occipital fasciculus, is observed. Patchy but symmetrical signal abnormalities are also seen in the thalami. No post-contrast enhancement is present neither in the brain nor in the cochlea. DWI shows increased mean diffusivity (more marked than in previous examinations). On ^1^H-MRS, mild decrease of the NAA peak in the white matter is present.

On CT performed in 2006, the white matter density was only slightly abnormal. Four years later, diffuse white matter hypodensity was present. Tiny subcortical calcifications, barely visible on the first CT scan, became more evident particularly in the frontal regions.

**Routine Laboratory Investigations**

The following investigations were performed *before treatment* with corticosteroids in order to rule out infectious and non-infectious causes of chronic meningitis and were all normal or negative:

- direct microscopic examination of cerebrospinal fluid (CSF) for bacteria, including *M. tuberculosis*;
- CSF culture for bacteria, including *M. tuberculosis*, and fungi;
- Serum and CSF VDRL for *Treponema pallidum*;
- *C. neoformans* capsular antigen;
- Serum and CSF antibodies anti-*Borrelia burgdorferi*, -*Brucella*, -*Toxoplasma gondii*, -HSV1-2, -VZV, -CMV, -EBV, -HIV, -rubeola virus, -morbillivirus;
- PCR for HSV1-2, VZV, CMV, EBV, HHV6, JCV, HIV, HTLV-I/II, *M. tuberculosis*, and *T. whippelii*;
- serum antibodies against and PCR for HHV8 (performed before treatment with tocilizumab);
- antibodies to double stranded DNA and ENA (performed also after treatment with corticosteroids and tocilizumab);
- antibodies to cardiolipin, thyroid, gliadin, endomysium, tissue transglutaminase, acquaporin-4, Hu, Yo, Ma2, CV2/CRMP5, Ri and amphiphysin antibodies;
- anti-neutrophil cytoplasmic antibodies (ANCA);
- lupus anticoagulant (LAC), rheumatoid factor, circulating immune complexes, C4, ACE, chitotriosidase, HLA-B51;
- serum and CSF vascular endothelial growth factor (VEGF);
- chest X-ray and whole-body CT with contrast;
- whole-body ^18^FDG-PET (performed before treatment with tocilizumab).

C3 was slightly increased (1.95 g/L, nv 0.8-1.7), and antinuclear antibodies (ANA) were negative, borderline or positive, with titers ranging from 1:40 to 1:640 (speckled). No abnormality was found in the study of lymphocyte subpopulations in blood (CD3+, CD4+, CD8+ and CD19+) and CSF (CD3+ and CD19+), except for a slight increase in the percentage of CD8+ lymphocytes.

**Motor and Somatosensory Evoked Potentials**

Motor evoked potentials (MEPs) (Oct-2009) recorded from *opponens pollicis* (OP) and *abductor hallucis* (AH) documented a slight increase in central motor conduction time only from the motor cortex to the lumbar segments of the spinal cord. Somatosensory evoked potentials (SEPs) (Oct-2009) were normal for the upper limbs, but documented a prolongation of the central conduction times from lower limbs.

**Visual and Brainstem Auditory Evoked Potentials**

Abnormal visual and brainstem auditory evoked potentials (VEPs and BAEPs) were in agreement with the presence of the optic neuropathy secondary to the long-lasting bilateral papilledema and to the progressive hearing loss, respectively.

**Array-CGH analysis**

Genome-wide array comparative genomic hybridization (CGH) analysis was performed using genomic DNA from patient peripheral blood (Gentra Puregene Blood Kit, Cat. no. 158467; Qiagen, [www.qiagen.com](http://www.qiagen.com/)) by a whole genome oligonucleotide microarray platform (Human Genome CGH 180K Microarray Kit; Agilent Techologies, Santa Clara, CA, USA) according to the manufacturer’s instructions. Images were acquired with a DNA microarray scanner (Agilent G2565BA) and data analysis was performed using the Agilent Feature Extraction software 9.1, the DNA Analytics software 4.0.76 DNA and the human haploid genomic reference DNA sequence (NCBI36/hg18) on the UCSC Human Genome browser.
