# Supplementary material for: An autoinflammatory neurological disease due to interleukin 6 hypersecretion
Source: J Neuroinflammation. 2013 Feb 21;10:29. doi: 10.1186/1742-2094-10-29 (PMC3601972; doi:10.1186/1742-2094-10-29)
Supplement: Additional file 2 — Figure showing IL-6 mRNA levels. [file 1742-2094-10-29-S2.doc]

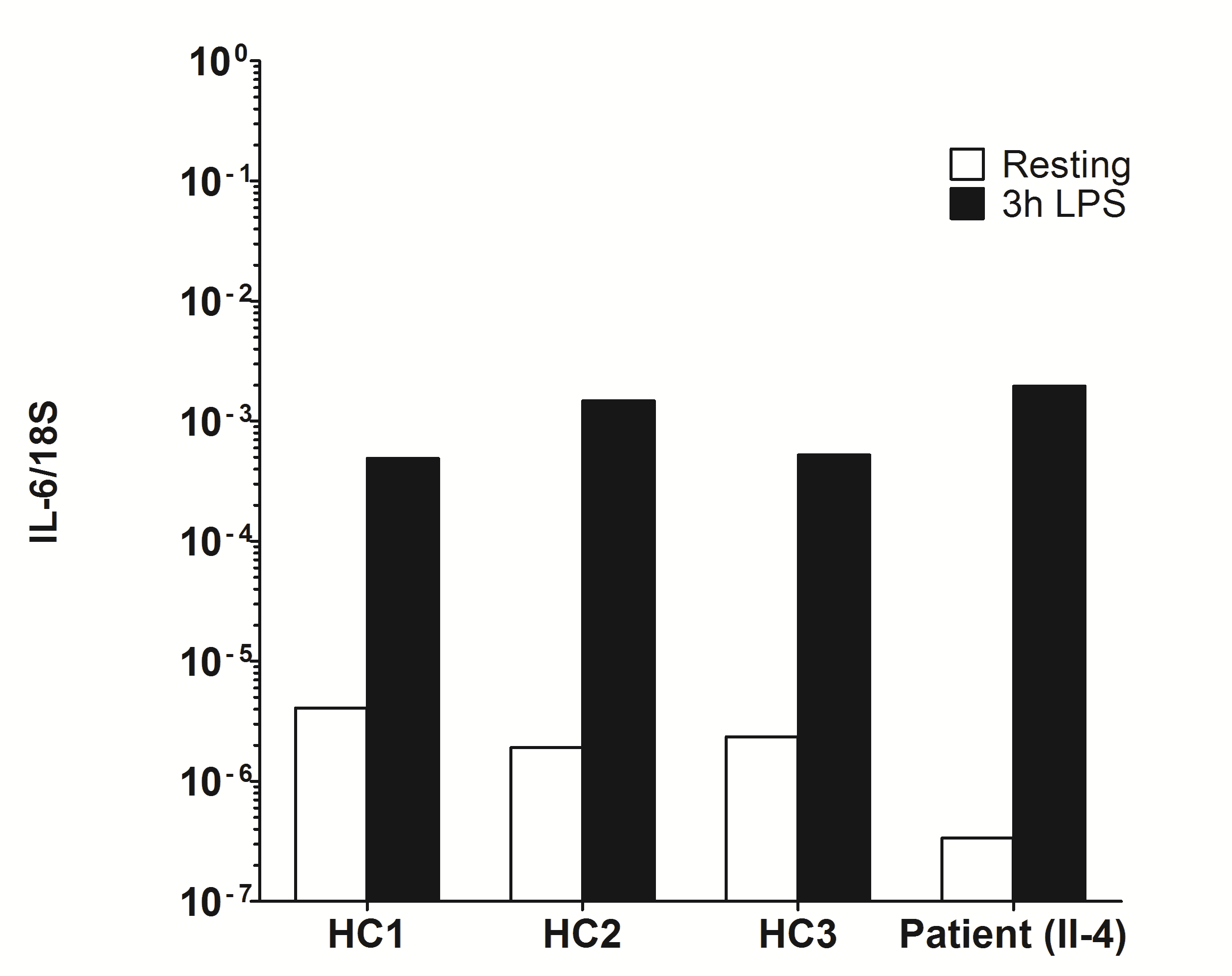


**Additional file 2 - Figure. IL-6 mRNA levels.** The levels of *IL-6* mRNA normalized by *18S* were determined in three healthy controls (HCs) and in the monocytes of the patient (II-4) after 18 hours *in vitro* (resting condition) and after further three hours of treatment with LPS (LPS). Compared to HCs, the amount of *IL-6* mRNA normalized by *18S* is lower in the monocytes of our patient under resting conditions, but it is similar after LPS treatment. Of note, we also found that the levels of *IL-6* mRNA directly isolated from the peripheral mononuclear blood cells (PMBCs) of our patient was lower than those of two healthy controls (data not shown), strongly suggesting that the high levels of circulating IL-6 are not derived from PBMCs.
