# Supplementary material for: An autoinflammatory neurological disease due to interleukin 6 hypersecretion
Source: J Neuroinflammation. 2013 Feb 21;10:29. doi: 10.1186/1742-2094-10-29 (PMC3601972; doi:10.1186/1742-2094-10-29)
Supplement: Additional file 3 — Figure showing soluble IL-6 receptor concentrations before and after tocilizumab. [file 1742-2094-10-29-S3.doc]

**
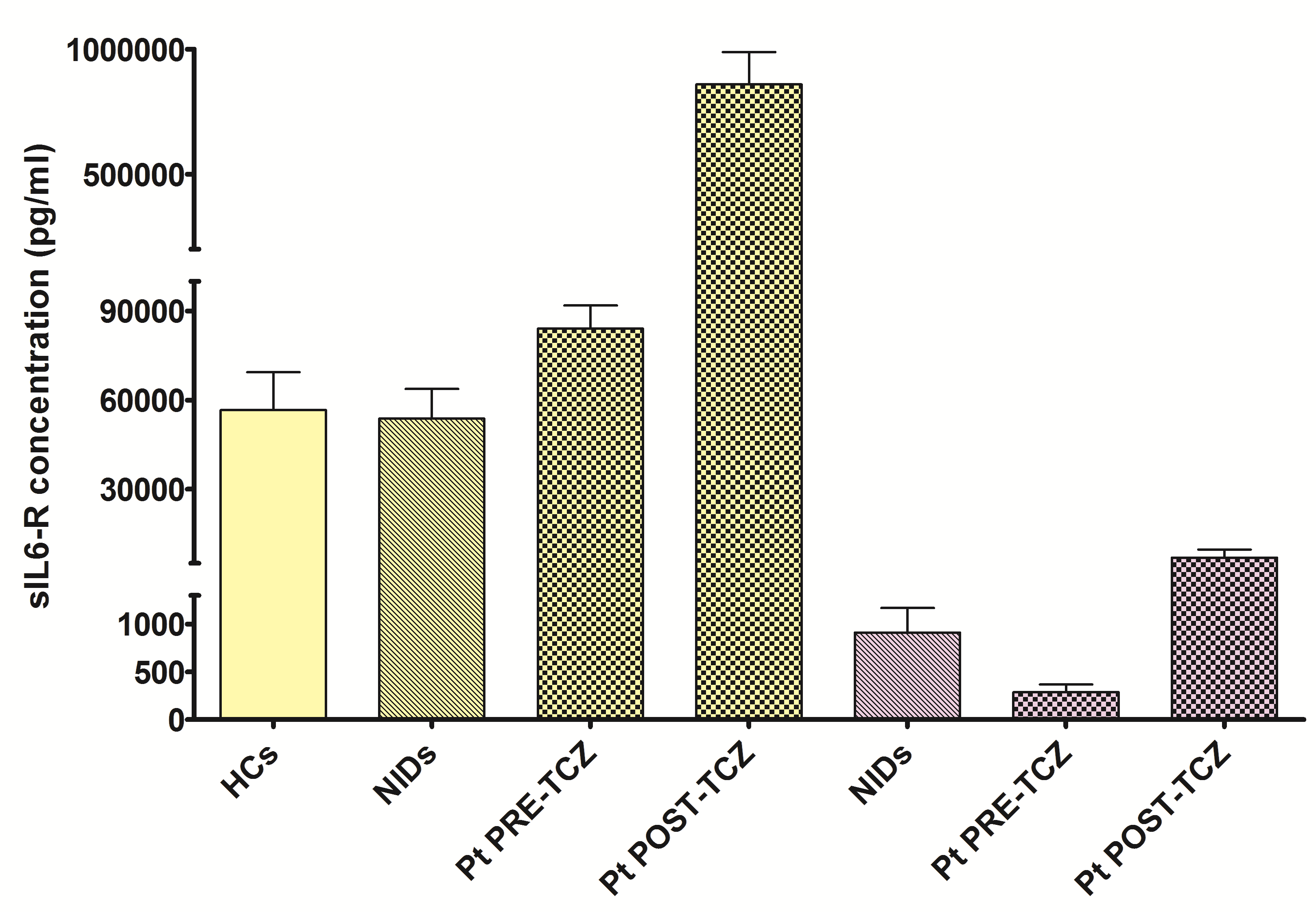
**

**Additional file 3 - Figure. Soluble IL-6 Receptor concentrations before and after tocilizumab**.

Soluble IL6-R (sIL6-R) concentration in the serum (yellow bars) of our patient before tocilizumab (Pt PRE-TCZ) is slightly higher than the sIL6-R concentration in the serum of five healthy controls (HCs) and nine patients with non-inflammatory diseases (NIDs) of the central nervous system (CNS). A marked increase of sIL6-R is observed after tocilizumab administration, and it is likely to be due to the prolongation of its elimination half-life by the formation of tocilizumab/sIL-6R immune complex. sIL6-R concentration in CSF (violet bars) of our patient (Pt PRE-TCZ) is very low compared with sIL6-R in serum, and it is slightly lower than the sIL6-R concentration in CSF of six control patients with NIDs of the CNS. A marked increase of CSF sIL6-R is observed after tocilizumab administration (Pt POST-TCZ), as occurred in the serum. For the patient, the values are the mean of at least three different experiments.
